# Evaluation of Sphingolipids in Wistar Rats Treated to Prolonged and Single Oral Doses of Fumonisin B_1_

**DOI:** 10.3390/ijms10010050

**Published:** 2008-12-27

**Authors:** Glória M. Direito, Adriana P. Almeida, Simone Aquino, Tatiana Alves dos Reis, Claudia Rodrigues Pozzi, Benedito Corrêa

**Affiliations:** 1 Departamento de Microbiologia e Imunologia, Instituto de Veterinária da Universidade Federal Rural do Rio de Janeiro, CEP 23890.000, Rio de Janeiro, RJ, Brasil. E-Mail: gdireito@ufrrj.br; 2 Departamento de Bromatologia e Química do Instituto Adolfo Lutz, CEP. 01246-902, São Paulo, Brasil. E-Mail: apalma@ial.sp.gov.br; 3 Instituto de Pesquisa Energéticas e Nucleares (IPEN / CNEN - SP), CEP 05508-000, São Paulo, SP, Brasil. E-Mail: siaq06@hotmail.com; 4 Departamento de Microbiologia do Instituto de Ciências Biomédicas da Universidade de São Paulo, CEP: 05508-900, São Paulo, SP, Brasil. E-Mail: tareis@usp.br; 5 Instituto de Zootecnia, Nova Odessa, 13460-000, SP, Brasil. E-mail: pozzi@iz.sp.gov.br

**Keywords:** Fumonisin B_1_, sphingolipids, biomarkers, Wistar rats, prolonged effect, single dose

## Abstract

The objective of the present study was to evaluate sphingolipid levels (sphingosine-So and sphinganine-Sa) and to compare the Sa/So ratio in liver, serum and urine of Wistar rats after prolonged administration (21 days) of fumonisin B_1_ (FB_1_). In parallel, the kinetics of sphingolipid elimination in urine was studied in animals receiving a single dose of FB_1_. Prolonged exposure to FB_1_ caused an increase in Sa levels in urine, serum and liver. The most marked effect on sphingolipid biosynthesis was observed in animals treated with the highest dose of FB_1_. Animals receiving a single dose of FB_1_ presented variations in Sa and So levels and in the Sa/So ratio.

## 1. Introduction

Fumonisins are a group of toxic metabolites produced by fungi of the genus *Fusarium*, especially *F. verticillioides (*Syn., *F. moniliforme*) and *F. proliferatum* [1, 2]. These species have been found in several agricultural products worldwide, especially in maize [3]. Among the fumonisins identified so far, fumonisin B_1_ (FB_1_) is considered to be the most toxic and the most abundant, representing ca. 70% of the total concentration in naturally contaminated food and feeds, followed by fumonisins B_2_ (FB_2_) and B_3_ (FB_3_). Ingestion of FB_1_ causes a variety of toxicosis in animals, including leukoencephalomalacia in horses [4], porcine pulmonary edema [5], and hepatocarcinoma and liver disease in rats [6]. Body weight and average daily weight gain have been shown to decrease in chicks in parallel with increasing dietary FB_1_ [7]. Hepatic and renal toxicity can be observed in several species, including rats, broilers, turkeys and ducks [8]. Additionally, the occurrence of FB_1_ in foods has been statistically associated with a high incidence of human esophageal cancer [9–11]. On the basis of existing toxicological evidence, the International Agency for Research on Cancer (IARC) has declared that *F. verticillioides* toxins are potentially carcinogenic to humans (Group 2B carcinogens) [12].

Due to its analogous structure to the precursor bases of sphingolipids, FB_1_ is able to block ceramide synthase, an enzyme that catalyzes the acylation of sphingosines (So) within the biosynthetic pathway of sphingolipids, causing immediate effects such as the depletion of complex sphingolipids, an increase in free sphinganines (Sa), or reduced reacylation of So [13–18]. Inhibition of ceramide synthase, results in an increase of cellular Sa concentration and, occasionally, a less pronounced increase of So, with a consequent increase in the Sa to So ratio [19]. Since the accumulated Sa cannot be completely metabolized, part of it is released into the extracellular medium, a fact that permits the detection of this compound in urine, tissue and blood [20–22]. These changes are observed before the occurrence of other biochemical indicators of cytotoxicity and the Sa/So ratio is thus an indicator or functional biomarker of exposure to toxic levels of fumonisin, although it is inefficient since fumonisins are eliminated from the organism immediately after ingestion [19, 22, 23]. Carcinogenicity is related to the accumulation of sphingoid bases (So and Sa) that trigger unprogrammed DNA synthesis, alterations in signaling mechanisms (cyclic AMP) and inhibition of protein kinase C, events culminating in the interruption of the normal cell cycle [24].

According to Shephard *et al*. [18] the elevation of Sa or of the Sa/So ratio is a useful biomarker in animal studies where exposures or doses are high. However, exposure in most human populations may be too low to produce marked changes in these parameters, which are naturally present in human physiological fluids. Solfrizzo *et al*. [20] concluded that, although the urine samples from areas with high maize consumption showed mean Sa/So ratio significantly higher as compared to low or no maize consumption areas, further studies is necessary before the Sa/Sa ratio can be considered a useful biomarker of fumonisin exposure in human.

According Pozzi *et al*. [25] the kidneys were affected by the joint action of AFB_1_ and FB_1_ in Wistar male rats and to some authors male rats are more susceptible than female rats to the nephrotoxic action of FB_1_. They observed that renal lesions appear to be more marked lower doses of FB_1_ in males, whereas the study was performed in Fischer 344 rats and B6C3F mice and kidney toxicity was studied in Sprague-Dawley rats [26–28]. The kinetics of fumonisin B_1_ after a single oral dose of 10 mg FB_1_/kg in male Wistar rats were studied by Martinez-Larranaga *et al*. [29] that observed almost 15 time more FB_1_ in the kidney than the liver. In view of the importance of investigations about biomarkers to fumonisin B_1_ exposure, the objective of the present study was to evaluate the levels of sphingolipids in urine, serum and liver of Wistar rats submitted to prolonged and single oral dose of fumonisin B_1_.

## 2. Materials and methods

### 2.1. Mycotoxins

Purified FB_1_ (>95%) administrated to rats was purchased from the Programme on Mycotoxins and Experimental Carcinogenesis (PROMEC, Tygerberg, South Africa).

### 2.2. Animals and diet

Fifty male Wistar rats with at 45 days of age a mean weight of 150 g, obtained from the Central Animal House of the State University of Campinas (UNICAMP), were studied. Thirty animals were used for the evaluation of the effects of prolonged exposure and 20 for the study of the effects of a single FB_1_ dose. The animals were divided into five groups and housed in cages of five animals each, with water and commercial chow being available *ad libitum*. In the case of animals submitted to prolonged exposure (21 days), urine, serum and liver tissue were collected before and 21 days after administration. In the case of animals used in the single dose assay, urine was collected at 24-hour intervals during the 96-hour administration period. The urine collection was performed by containment and by spontaneous urination. The diets especially prepared for rats by Nuvital Nutrients S/A were analyzed for the presence of aflatoxinas, ochratoxins, sterigmatocystin, zearalenone [30] and fumonisins [31]. The study was approved by the Institutional committee on the Care and Use of Laboratory Animals of the University of São Paulo.

### 2.3. Experimental protocol

All animals received FB_1_ solution in sterile saline by gavage, and were sacrificed under anesthesia with 3% sodium pentobarbital. The animals were divided into the following experimental groups: *Prolonged exposure*: the animals were divided into three groups – control group I (GI - no mycotoxins – control), group II (GII - 5 mg FB_1_/kg b.w. /day), and group III (GIII - 10 mg FB_1_/kg b.w. /day); *Single dose*: the animals were divided into two groups: group I (no mycotoxins – control) and group II (5 mg FB_1_ /kg b.w).

### 2.4. Sphingolipid analysis

The total amounts of sphingosine (So) and sphinganine (Sa) in liver, serum and urine were determined as described by Riley *et al*. [32] and Castegnaro *et al*. [33, 34]. The extracts were derivatized with reactive OPA and analyzed by HPLC analysis on a Supelcosil LC18 reverse-phase column according to Shephard and Van Der Westhuizen [35] using a mobile phase of methanol and water (9:1, v/v) at a flow rate of 1 mL/min. Sphinganine (dl-erythrodihydrosphingosine) and sphingosine (d-sphingosine), used as standards, were obtained from Matreya Inc. The amounts of sphinganine and sphingosine were calculated using the relative areas of the unknowns vs the C_20_ sphinganine internal standard.

### 2.5. Statistical analysis

Analysis consisted of a 2 × 2 factorial scheme in a fully randomized design, with four replicates calculated on a case by case basis. Analysis of variance (Tukey test) was performed combining the treatments used with the liver, serum and urine samples by order.

## 3. Results

### 3.1. Prolonged effect

The prolonged administration of FB_1_ (groups II and III) resulted in an increase of Sa levels in urine, serum and liver or rats treated with the toxin compared to the control group. In addition, mean Sa levels were higher than the observed So concentrations. The highest concentrations of Sa were detected in serum (38.03 ng/mL), liver (27.73 ng/mL) and urine (23.54 ng/mL) of group III animals administrated with the highest FB_1_ dose (10 mg/kg). With respect to So, the highest levels were observed in rats treated with the lower FB_1_ dose (5 mg/kg), especially in serum (24.53 ng/mL). The highest Sa/So ratios were observed in urine (8.03) of group III animals inoculated with the higher FB_1_ concentration (Table 1).

### 3.2. Single dose

Mean urinary So concentrations were below the values observed in the first sampling (T0) in animals receiving a single dose of FB_1_ (5 mg/kg b.w.). In contrast, Sa levels showed a significant increase (p<0.05) 48 hours after the administration of the toxin (13.39 ng/mL), reaching a maximum level at 72 hours (30.46 ng/mL) (Table 2). The same was observed for the Sa/So ratio, which reached maximum values at 72 hours (3.03) and tended to return to normal at the end of the study period (0.68). Mean Sa/So ratio in rats receiving a single dose of FB_1_ ranged between 0.57 to 3.03.

Sa and So concentrations remained constant (p>0.05) in the control group (G I) throughout the study period with Sa/So ratio ranging from 0.57 to 0.63 with value close to those observed in T0.

## 4. Discussion

Rats have been used over decades as an experimental model in mycotoxicology studies, especially investigations on aflatoxins [24], and were also employed in the first studies on fumonisins conducted by Gelderblom *et al*. [1] in South Africa. The choice of the use of male rats in the present study was based on previous investigations demonstrating a higher sensitivity of males to the effects of fumonisins compared to females [36]. Furthermore, Riley *et al*. [13] studying differences in FB_1_ sensitivity between sexes in Sprague-Dawley rats, observed higher Sa levels and a higher Sa/So ratio in male rats compared to female animals. Differences in the responses to FB_1_ between sexes of the same animal strain have also been demonstrated by Tolleson *et al*. [37]. In order to characterize the effect of natural intoxication on the synthesis of sphingolipids, we chose to administer the toxin by the oral route over 21 days. The FB_1_ doses were selected based on the literature in which the most notable effects of FB_1_ were observed in liver, bone marrow, adrenal glands and kidneys, with doses ranging from 1 to 75 mg FB_1_/kg b.w./day administered for 14 days [38, 39].

The effects of FB_1_ have been investigated since its discovery in 1988 by Gelderblom *et al*. [1]. Although studies in this area are still incipient, the observation of the blockade of sphingolipid synthesis has clarified the origin of some lesions and physiological alterations resulting from FB_1_ intoxication [40].

FB_1_, by reacting with ceramide synthase (*N*-acyltransferase), the key enzyme of the sphingolipid biosynthetic pathway, triggers various signaling mechanisms for cell damage [41], causing the accumulation of immediate ceramide precursors, mainly Sa which is no longer acylated after its formation. The increase in the concentration of this precursor triggers a chain of events that are aimed at eliminating this substance which has become harmful to the cells, with the production of sphingosin-1-phosphate as the main product functioning as a secondary intracellular messenger that regulates the mobilization of calcium and cell survival. Thus, the dynamic equilibrium between the sphingolipid metabolites ceramide and sphingosine-1-phosphate, which regulate opposite signaling mechanisms, is an important factor in the determination of cell survival or cell death [21]. Under normal conditions, these degradation reactions are efficient. However, in some situations FB_1_ blocks ceramide synthase and metabolization of the accumulated Sa, which is continuously produced. As a compensatory mechanism, Sa is released into the extracellular medium, which permits its detection in urine and blood. Increased concentrations of free sphingoid bases in serum, urine, liver and kidneys have been reported to be correlated with the severity of hepatotoxicity, nephrotoxicity or other indicators of cytotoxicity [40, 42].

This fact was also demonstrated in the present experiment in which an increase of Sa levels was observed in urine, serum and liver, especially in animals receiving the highest dose of the toxin (10 mg FB_1_/kg b.w./day, group III). Although urinary Sa levels (23.54 ng/mL) were below those detected in serum (38.03 ng/mL) and liver (27.73 ng/mL) in group III and the urinary Sa/So ratio was more pronounced (8.03). According to these data is possible to observe that the kidney is a potential target organ indicating a possible nephrotoxic effect of FB_1._ The accumulation of Sa and the increased urinary Sa/So ratio closely reflected the changes that occurred in the kidney, the organ most sensitive to fumonisin-induced sphingolipid alterations, as defined by Riley *et al*. [13]. In male Sprague-Dawley rat and Fischer 344N rats, the accumulation of sphingoid bases and toxicity in kidney are much greater than in liver, at the same dose [28].

Garren *et al*. [39] found no detectable alterations in the kidney Sa/So ratio in rats receiving doses of up to 0.5 mg FB_1_/kg b.w., whereas doses ranging from 1.0 to 5.0 mg/kg had an effect on the kidney but no apparent dose-response was observed (p < 0.0001), with the increase in the Sa/So ratio in the kidneys not reflecting changes in the urinary Sa/So ratio (3.3 to 4.5).

Approximate urinary Sa/So ratios were observed in the present study when a dose of 5 mg FB_1_/kg was administered (2.53), whereas the dose of 10 mg/kg caused a 3-fold increase in the Sa/So ratio (8.03) (Table 1). Wang *et al*. [42] reported a significant increase of the Sa/So ratio in both kidney and urine of rats fed approximately 0.25 to 0.5 mg FB_1_/kg per day, while a diet containing 5 times lower FB_1_ levels resulted in a 2-fold increase of the Sa/So ratio in the kidney but no increase in urine. With respect to reversibility, the same author showed that in rats fed a dose of 10 μg FB_1_/g the levels of sphingoid bases returned to normal within a period of 10 days. Similar results have been reported by Garren *et al*. [39] who analyzed alterations in the Sa/So ratio in rat kidneys after the end of treatment with FB_1_.

An elevated Sa/So ratio has also been detected in the liver, the organ apparently most affected by FB_1_ [43]. In the present study, prolonged administration of FB_1_ demonstrated a direct relationship between the doses of 5 and 10 mg/kg (groups II and III) and an increase in liver Sa/So ratio, with the higher dose causing a 2-fold increase (2.90 and 5.80, respectively), thus demonstrating the dose-response sensitivity of this organ. The effect of FB_1_ on the biosynthesis of sphingolipids, as demonstrated by an increase of Sa levels and of the Sa/So ratio in urine, serum and liver, has also been observed by other investigators studying other species such as mammals and birds [44]. Different routes and frequencies of administration of FB_1_ influence the dose response in different species. Studies on rabbits have shown that the serum and urinary Sa/So ratio increased markedly compared to the control groups after intravenous administration of 1 mg FB_1_/kg w.b., for 5 days [45] and by gavage for 21 days [46]. On the other hand, the same dose administered by gavage to rats five times per week for 5 weeks resulted in no significant increase in the serum Sa/So ratio. Furthermore, only a marginal increase in serum Sa/So ratio was observed in rats receiving 16.8 mg FB_1_/kg b.w. three times per week by gavage [32].

Our results demonstrated a 3 and 4.5 times lower serum Sa/So ratio, compared to the ratios found in urine and liver, respectively, even when the maximum dose (10 mg FB_1_/kg) was administered for 21 days. Progressive increases in Sa and in the Sa/So ratio can be considered to be safe biomarkers for the evaluation of the effects of FB_1_ [48], even in the case of exposure to small doses. In rats, determination of the Sa/So ratio in urine and liver was found to be more sensitive than its determination in serum, probably due to the transitory state of the sphingoid bases in this material as a result of metabolization and excretion in rats. In contrast, Van der Westhuizen [47] demonstrated in monkeys that the plasma Sa/So ratio is a more sensitive biomarker of exposure to fumonisins than the urinary ratio. Urine levels of Sa/So ratio were more useful than serum levels in this present study. In despite of high levels of So and Sa in serum, the Sa/So levels were below in group II and III comparing to values of urine. In our experiment, the standard deviation of Sa/So level in serum very large can be attributed the low efficiency of extraction, fact indicated by Riley *et al*. [32], and also due to the majority of absorbed FB_1_ might bind to serum lipids or proteins, making undetectable as the free form [48]. The detected values of So, Sa, Sa/So in liver and urine were similar but urinary Sa/So ratio increased markedly compared to Sa/So in liver, as was observed in the animals that received 10 mg/kg b.w. Because of these difficulties, urine showed to be the best material for detection Sa/So ratio in Wistar rats.

Administration of a single dose of FB_1_ (5 mg/kg b.w.) provoked changes in urinary Sa, So and Sa/So ratio within the first 24 hours. Maximum Sa levels (30.46 ng/mL) were observed after 72 hours, followed by a marked decline (5.87 ng/mL) at the end of the study period (96 hours), with the levels being below those detected at T0 (6.05 ng/mL). The Sa/So ratio showed a 3-fold increase in the first 24 hours compared to baseline (from 0.57 to 1.90). After the ratio reached a maximum level at 72 hours (3.03), a marked reduction was observed until the end of the study period (96 hours), with the ratio tending to return to normal (0.68). In rats, many studies have showed the reversibility of damage in liver and kidney tissue induced by fumonisin [50]. The average of So values in single dose (24, 48, 72 and 96 hours) was 7.39 ng/mL and in comparison with prolonged dose after 21 days (7.08 ng/mL) the data showed a similar tendency in the decrease of So in urine. There are few reports that discuss the kinetic of Sa/So ratio in rats exposed to a single dose of FB_1_, rendering difficult the comparative analysis with our study. Cai *et al*. [49] studying time course changes in urinary Sa/So, in F344 rats treated with single-dose FB_1_ (10 and 25 mg/kg), observed that urinary Sa/So began to increase at 12 h after administration, continually increased, reached a maximum on days 5 and 7, respectively, and decreased thereafter. The urinary Sa/So of rats in the high-dose group was significantly higher than of the low-dose group at days, 5,7 and 10, showing dose-dependent increases of Sa/So and this fact was also observed in some studies [32,39], according to our results that showed similar tendency of Sa/So, using low doses of FB_1_ (5 mg/kg).

In the present study, urinary Sa/So also began to increase at 12 h after administration, continually increased, reached a maximum at 72 h. The interval between the maximum peak and decline in the Sa/So ratio was 24 hours and the return to normal within 96 h. Van der Westhuizen *et al*. [47], studied the effects of a single dose of FB_1_ (1 and 10 mg/kg) on Sa and So levels in vervet monkeys. The authors also observed a maximum peak in urinary Sa/So ratios at 72 hours, followed by a gradual decrease up to 120 hours, but the initial decline occurred in 96 hours. Cai *et al*. [49] and Delongchamp and Yong [51] reported that it is uncertain if the results obtained in rats can be extrapolated to larger animals since FB_1_ reported that the FB_1_ are rapidly eliminated from rats and mice while elimination in humans is believed to be slower.

Garren *et al*. [39] observed a rapid decrease of the Sa/So ratio in rat kidney after cessation of daily exposure to 1 mg FB_1_/kg b.w. within 24 h after the end of treatment, but the Sa/So ratio only returned to normal after a period of 1 to 3 weeks.

## 5. Conclusions

Comparison of prolonged (group II) and single-dose treatment with FB_1_ revealed a similar mean urinary Sa/So ratio (2.53 and 3.03, respectively). This finding indicates an almost constant excretion rate of sphingoid bases, irrespective of the duration of treatment when the same dose was administered (5 mg FB_1_/kg in both cases). In addition, no marked differences in urinary So levels were observed between group II (prolonged treatment) and animals receiving a single dose, with So levels showing a slight decline over time (Table 1 and 2). This finding confirms that Sa is responsible for the increase in the Sa/So ratio as reported in the literature; however, in the present study this effect was observed both after prolonged exposure and after administration of a single dose of FB_1_. Urine was found to be a good material for the determination of the Sa/So ratio in male Wistar rats compared to liver and serum since higher urinary levels are observed with increasing doses (group III, 10 mg FB_1_/kg). Van der Westhuizen *et al*. [47] working with vervet monkeys, dosed with 10 mg FB_1_/kg body weight, in a single dose, observed that the urinary Sa/So ratios showing an earlier increase than the serum, peaked after two days (72 h) and rapidly declined thereafter to pre-dosing levels after four days. However, the urinary ratio returned to its original level within the seven-day experimental period. Cai *et al*. [49] showed that in rats F344, dosed with the same dose, a maximum of Sa/So in urine at five day (120 hours), but the authors discussed that there are limited published data on Sa/So kinetics in experimental animals treated with single-dose FB_1_ to compare with their results. Our findings are significant, as observed in Wistar rats, which results approached to the monkeys data, receiving 5 mg FB_1_/kg b.w. besides, the kinetics of Sa/So showed that adverse effects are reversible after 72 h, as was observed in a single dose. The present work showed that male Wistar rats strains are good model to study about biosynthetic pathway of sphingolipids, and we suggest these animals to future researches about synergic effects of FB_1_ alone and in combination with other mycotoxins to evaluate the detectable threshold limit of Sa/So and the time of reversibility in urine.

## Figures and Tables

**Table 1.** Mean sphingosine (So) and sphinganine (Sa) levels and mean Sa/So ratio in urine, liver and serum or rats submitted to prolonged exposure (21 days) to FB_1_.

Material	Group	So (ng/mL)	Sa (ng/mL)	Sa/So
Urine (n=10)	I	**13.01^a^** (± 4.27)	**7.30^c^** (± 3.18)	**0.56^c^** (± 0.19)
	II	**7.08^c^** (± 1.25)	**17.95^ab^** (± 0.57)	**2.53^b^** (± 0.52)
	III	**2.93^c^** (± 1.25)	**23.54^a^** (± 8.28)	**8.03^a^** (± 1.35)
Serum (n=10)	I	**9.12^c^** (± 3.93)	**3.09^c^** (± 3.12)	**0.34^c^** (± 0.21)
	II	**24.53^a^** (±11.49)	**34.49^a^** (± 21.59)	**1.40^b^** (± 0.24)
	III	**21.67^a^** (± 21.25)	**38.03^a^** (± 26.59)	**1.75^a^** (± 4.83)
Liver (n=10)	I	**9.20^b^** (± 3.82)	**1.67^c^** (± 0.84)	**0.18^c^** (± 0.12)
	II	**4.81^c^** (± 2.91)	**13.85^b^** (± 6.73)	**2.90^b^** (± 1.56)
	III	**4.79^c^** (± 1.82)	**27.73^a^** (± 8.71)	**5.80^a^** (± 2.43)

Group I: control; group II: 5 mg FB_1_/kg b.w.; group III: 10 mg FB_1_/kg b.w.

Means followed by the same letters in the same column, in each clinical material, did not differ significantly by the Tukey test (p < 1%).

**Table 2.** Mean sphingosine (So) and sphinganine (Sa) levels and urine Sa/So ratio in rats receiving a single dose of FB_1_.

Time (hours)	So	Sa	Sa/So
0	10.52^a^ ±4.70*	6.05^c^ ±0.87	0.57^c^ ±0.24
24	3.55^c^ ±1.97	6.64^c^ ±2.87	1.9^ab^ ±0.89
48	7.34^b^ ±0.70	13.39^b^ ±1.39	1.82^ab^ ±0.33
72	10.03^a^ ±3.12	30.46^a^ ±9.88	3.03^a^ ±0.72
96	8.64^b^ ±4.77	5.87^c^ ±0.73	0.68^c^ ±0.83

n = 10 animals.

Group II: 5 mg FB_1_/kg b.w./day.

Sa and So are reported as ng/mL.

Means followed by the same letters in the same column did not differ significantly by the Tukey test (p >0.01).

## References

[1] Gelderblom WCA, Jaskiewicz K, Marasas WFO, Thiel PG, Horak RM, Vleggaar R, Kriek NPJ (1988). Fumonisins — Novel mycotoxins with cancer promotion activity produced by *Fusarium moniliforme*. Appl. Environ. Microbiol.

[2] Nelson PE, Plattner RD, Shackelford DD, Desjardins AE (1992). Fumonisin B_1_ production by *Fusarium* species other than *F. moniliforme* in Section Liseola a by some related species. Appl. Environ. Microbiol.

[3] Marasas WFO, Kellerman TS, Gelderblom WCA, Coetzer JAW, Thiel PG, Van Der Lugt JJ (1988). Leukoencephalomalacia in horse induced by fumonisin B_1_ isolated from *Fusarium moniliforme*. Onderstepoort J. Vet. Res.

[4] Kellerman TS, Marasas WFO, Theil PG, Gelderblom WCA, Cawood M, Coetzer JAW (1990). Leukoencephalomalacia in two horses induced by oral dosing of fumonisin B_1_. Onderstepoort J. Vet. Res.

[5] Dilkin P, Zorzete P, Mallmann CA, Gomes JDF, Utiyama CE, Oetting LL, Corrêa B (2003). Toxicological effects of chronic low doses of aflatoxin B_1_ and fumonisin B_1_ containing *Fusarium moniliforme* culture material in weaned piglets. Food Chem. Toxicol.

[6] Gelderblom WCA, Lebepe-Mazur S, Snijman PW, Abel S, Swanevelder S, Kriek NPJ, Marasas WFO (2001). Toxicological effects in rats chronically fed low dietary levels of fumonisin B_1_. Toxicology.

[7] Ledoux DR, Brown TP, Weibking TS, Rottinghaus GE (1992). Fumonisin toxicity in broiler chicks. J. Vet. Diagn. Invest.

[8] Tran ST, Tardieu D, Auvergne A, Bailly JD, Babilé R, Durand S, Benard G, Guerre P (2006). Serum sphinganine and the sphinganine to sphingosine ratio as a biomarker of dietary fumonisins during chronic exposure in ducks. Chem. Biol. Interact.

[9] Rheeder JP, Marasas WFO, Theil PG, Sydenham EW, Shephard GS, Van Schalkwyk DJ (1992). *Fusarium moniliforme* and fumonisins in corn in relation to human esophageal cancer in Transkei. Phytopathology.

[10] Sydenham EW, Theil PG, Marasas WFO, Shephard GS, Vanschalkwyk DJ, Koch KR (1990). Natural occurrence of some *Fusarium* mycotoxins in corn from low and high esophageal cancer prevalence areas of the Transkei, Southern Africa. J. Agric. Food Chem.

[11] Marasas WFO, Jackson LS, de Vries JW, Bullerman LB (1996). Fumonisins: History, World-wide Occurrence and Impact.

[12] International Agency for Research on Cancer (IARC) (1993). Toxins Derived from Fusarium Moniliforme: Fumonisins B_1_ and B_2_ and Fusarin C (Group 2B), Monographs on the Evaluation of Carcinogenic Risks to Humans.

[13] Riley RT, Hinton DM, Chamberlain WJ, Bacon CW, Wang E, Merrill AH, Voss K (1994). Dietary fumonisin B_1_ induces disruption of sphingolipid metabolism in Sprague-Dawley rats: A new mechanism of nephrotoxicity. J. Nutr.

[14] Riley RT, Wang E, Schroeder JJ, Smith ER, Plattner RD, Abbas H, Yoo HS, Merrill AH (1996). Evidence for disruption of sphingolipid metabolism as a contributing factor in the toxicity and carcinogenicity of fumonisins. Nat. Toxins.

[15] Riley RT, Voss KA, Norred WP, Sharma RP, Wang E, Merrill AH (1998). Fumonisins: Mechanism of mycotoxicity. Revue Méd. Vét.

[16] Merrill AH, Wang E, Vales TR, Smith ER, Schroeder JJ, Menaldino DS, Alexander C, Crane HM, Xia J, Liotta DC, Meredith FI, Riley RT, Jackson LS, de Vires JW, Bullerman LB (1996). Fumonisin Toxicity and Sphingolipid Biosynthesis..

[17] Turner PC, Nikiema P, Wild CP (1999). Fumonisin contamination of food progress in development of biomarkers to better assess human health risks. Mutat. Res.

[18] Shephard GS, Van Der Westhuizen L, Sewram V (2007). Biomarkers of exposure to fumonisin mycotoxins: A review. Food Addit. Contam.

[19] Ribar S, Mesaric M, Bauman M (2001). High-performance liquid chromatographic determination of sphinganine and sphingosine in serum and urine of subjects from an endemic nephropathy area in Croatia. J. Chromatogr. B.

[20] Solfrizzo M, Visconti A, Avantaggiato G, Torres A, Chulze S (2000). *In vitro* and *in vivo* studies to assess the effectiveness of cholestiramine as a binding agent for fumonisins. Mycopathologia.

[21] Spiegel S, Milstien S (2000). Sphingosine-1-phosphate: signaling inside and out. FEBBS Letters.

[22] Shephard GS, Theil PG, Sydenham EW, Savard ME (1995). Fate of a single dose of ^14^C-labeled fumonisin B_1_ in vervet monkeys. Nat. Toxins.

[23] Wang E, Ross F, Wilson TM, Riley RT, Merrill AH (1992). Increase in serum sphingosine and sphinganine and decrease in complex sphingolipids in ponies given feed containing fumonisins, mycotoxins produced by *Fusarium moniliforme*. J. Nutr.

[24] Hussein HS, Brasel JM (2001). Toxicity, metabolism and impact of mycotoxin on humans and animals. Toxicology.

[25] Pozzi CR, Corrêa B, Xavier JG, Direito GM, Orsi RB, Matarazzo SV (2000). Effects of prolonged oral administration of fumonisin B_1_ and aflatoxin B_1_ in rats. Mycopathologia.

[26] Voss KA, Chamberlain WJ, Bacon CN, Alberts RA, Walters DB, Norred WP (1995). Subchronic feeding study of the mycotoxin fumonisin B_1_ in B6C3F mice and Fischer 344 rats. Fund. Appl. Toxicol.

[27] Voss KA, Howard PC, Riley RT, Sharma RP, Bucci TJ, Lorentzen RJ (2002). Carcinogenicity and mechanism of action of fumonisin B_1_: a mycotoxin produced by *Fusarium moniliforme* (= *F. verticillioides*). Cancer Det. Prev.

[28] Riley RT, Voss KA (2006). Differential sensitivity of rat kidney and liver to fumonisin toxicity: Organ-specific differences in toxin accumulation and sphingoid base metabolism. Toxicol. Sci.

[29] Martinez-Larranaga MR, Anadon A, Diaz MJ, Fernandez-Cruz ML, Martinez MA, Frejo MT, Martinez M, Fernandez R, Anton RM, Morales ME (1999). Toxicokinetics and oral bioavailability of fumonisin B_1_. Vet. Hum, Toxicol.

[30] Soares LMV, Rodrigues-Amaya D (1989). Survey of aflatoxins, ochratoxin A, zearalenone and sterigmatocystin in some Brazilian foods by using multitoxin thin layer chromatographic method. J. Assoc. Off. Anal. Chem.

[31] Sydenham EW, Shephard GS, Thiel PG (1996). Liquid Chromatographic determination of fumonisins B_1_, B_2_ and B_3_ in corn. J. Assoc. Off. Anal. Chem.

[32] Riley RT, Wang E, Merrill AH (1994). Liquid chromatographic determination of sphinganine and sphingosine: Use of the free sphinganine-to-sphingosine ratio as a biomarker for consumption of fumonisins. AOAC Int.

[33] Castegnaro M, Garren L, Galendo D, Gelderblom WCA, Chelule P, Dutton MF, Wild CP (1998). Analytical method for the determination of sphinganine and sphingosine in serum as a potential biomarker for fumonisin exposure. J. Chromatogr. B.

[34] Castegnaro M, Garren L, Gaucher I, Wild CP (1996). Development of a new method for the analysis of sphinganine and sphingosine in urine and tissues. Nat. Toxins.

[35] Shephard GS, van der Westhuizen L (1998). Liquid chromatographic determination of the sphinganine/sphingosine ratio in serum. J. Chromatogr. B.

[36] Voss KA, Plattner RD, Riley RT, Meredith FI, Norred WP (1998). *In vivo* effects of fumonisin B_1_-producing and fumonisin B_1_-nonproducing *Fusarium moniliforme* isolates are similar: Fumonisins B_2_ and B_3_ cause hepato and nephrotoxicity in rats. Mycopathologia.

[37] Tolleson WH, Dookey KL, Sheldon WG, Thurman JD, Bucci TJ, Howard PC, Jackson LS, De Vries JW, Bullerman LB (1996). The Mycotoxin Fumonisin Induces Apoptosis in Cultures Human Cells and Livers and Kidneys of Rats.

[38] Bondy GS, Suzuki CAM, Fernie SM, Armstrong CL, Hierlihy SL, Savard ME, Barker MG (1997). Toxicity of fumonisin B_1_ to B6C3F_1_ mice: A 14 day gavage study. Food Chem. Toxicol.

[39] Garren L, Galendo D, Wild CP, Mastegnaro M (2001). The induction and persistence of altered sphingolipid biosynthesis in rats treated with fumonisin B_1_. Food Addit. Contam.

[40] Riley RT, Enongene E, Voss KA, Norred WP, Meredith FI, Sharma RP, Spitsbergen J, Williams DE, Carlson DB, Merrill AH (2001). Sphingolipid perturbation as mechanism for fumonisin carcinogenesis. Environ. Health Perspect.

[41] Merrill AH, Schmelz EM, Dillehay DL, Spiegel S, Shayman JA, Schroeder JJ, Rilley RT, Voss KA, Wang E (1997). Sphingolipids — The enigmatic lipid class; Biochemistry, Physiology and Pathophysiology. Toxicol. Appl. Pharmacol.

[42] Wang E, Riley RT, Meredith FI, Merrill AH (1999). Time course dependence, and reversibility of increases in urinary sphinganine and sphingosine in animals fed defined diets containing fumonisin B_1_: Characteristics of urinary biomarkers for exposure to fumonisins. J. Nutr.

[43] Enongene EN, Sharma RP, Bhandari N, Voss KA, Riley RT (2000). Disruption of sphingolipid metabolism in small intestines, liver and kidney of mice dosed subcutaneously with fumonisin B_1_. Food Chem. Toxicol.

[44] Tran ST, Bailly JD, Tartdieu D, Durand S, Benard G, Guerre P (2003). Shinganine to sphingosine ratio and predictive biochemical markers of fumonisin B_1_ exposure in ducks. Chem. Biol. Interact.

[45] Gumprecht LA, Marcucci A, Weigel RM, Vesonder RF, Riley RT, Showker JL, Beasley VR, Haschek WM (1995). Effects of intravenous Fumonisin B_1_ in rabbits: Nephrotoxicity and sphingolipid alterations. Nat. Toxins.

[46] Laborde JB, Terry KK, Howard PC, Chen JJ, Collins TF, Shackelford ME, Hansen DK (1997). Effects of intravenous Fumonisin B_1_ in rabbits: Nephrotoxicity and sphingolipid alterations. Fundam. Appl. Toxicol.

[47] Van der Westhuizen L, Shephard GS, Van Schalkwyk DJ (2001). The effect of a single gavage dose of fumonisin B_2_ on the sphinganine and sphingosine concentrations in vervet monkeys. Food Chem. Toxicol.

[48] Soriano JM, González L, Catalá AI (2005). Mechanism of action of sphingolipids and their metabolites in the toxicity of fumonisin B_1_. Progr. Lipids Res.

[49] Cai Q, Tang L, Wang JS (2007). Validation of fumonisin biomarkers in F344 rats. Toxicol. Appl. Pharmacol.

[50] Voss KA, Riley RT, Norred WP, Bacon CW, Meredith FI, Howard PC, Plattner RD, Collins TFX, Hansen DK, Porter JK (2001). An overview of rodent toxicities: Liver and kidney effects of fumonisins and *Fusarium moniliforme*. Environ. Health Perspect.

[51] Delongchamp RR, Young JF (2001). Tissue sphinganine as a biomarker of fumonisin-induced apoptosis. Food Addit. Contam.

